# Cycling in primary progressive multiple sclerosis (CYPRO): study protocol for a randomized controlled superiority trial evaluating the effects of high-intensity interval training in persons with primary progressive multiple sclerosis

**DOI:** 10.1186/s12883-023-03187-6

**Published:** 2023-04-22

**Authors:** Marie Kupjetz, Niklas Joisten, Annette Rademacher, Roman Gonzenbach, Jens Bansi, Philipp Zimmer

**Affiliations:** 1 Department of Performance and Health (Sports Medicine), Institute of Sport and Sport Science, TU Dortmund University, Otto-Hahn-Straße 3, 44227 Dortmund, Germany; 2 Marianne-Strauß-Klinik, Behandlungszentrum Kempfenhausen für Multiple Sklerose Kranke gGmbH, Milchberg 21, 82335 Berg, Germany; 3 Department of Neurology, Clinics of Valens, Rehabilitation Centre Valens, Taminaplatz 1, 7317 Valens, Switzerland; 4 Department of Health, OST – Eastern Switzerland University of Applied Sciences, Rosenbergstrasse 59, 9001 Sankt Gallen, Switzerland

**Keywords:** Multiple sclerosis, Primary progressive multiple sclerosis, High-intensity interval training, Physical fitness, Cardiorespiratory fitness, Serum neurofilament light chain, Glial fibrillary acidic protein, Kynurenine pathway

## Abstract

**Background:**

Primary progressive multiple sclerosis (PPMS) is the least prevalent multiple sclerosis (MS) phenotype. For persons with PPMS (pwPPMS), pharmacological treatment options are limited. As a complementary non-pharmacological treatment, endurance training improves the health-related quality of life (HRQoL), numerous MS symptoms, and MS-related performance impediments. High-intensity interval training (HIIT) has been shown to induce superior effects compared to moderate-intensity continuous training (MCT). As current evidence is based on MS samples with mixed phenotypes, generalizability to pwPPMS remains unclear.

**Methods:**

CYPRO is a parallel-group, single-center, and single-blind randomized controlled superiority trial evaluating the effects of HIIT compared to MCT in pwPPMS. Sixty-one pwPPMS are randomized (1:1) to perform volume-matched HIIT or MCT sessions on bicycle ergometers two to three times per week in addition to standard rehabilitative care during their three-week inpatient stay at Valens rehabilitation clinic, Switzerland. Standard rehabilitative care comprises endurance and strength training, physiotherapy, and occupational therapy. HIIT sessions include six 90-second intervals at 95% peak heart rate (HR_peak_), interspersed by 90-second active breaks with unloaded pedaling, aimed to reach 60%HR_peak_. MCT represents the standard treatment at Valens rehabilitation clinic and is performed as continuous cycling at 60%HR_peak_ for the duration of 26 minutes. The primary outcome is cardiorespiratory fitness, assessed as peak oxygen consumption (V̇O_2peak_) during cardiopulmonary exercise testing (CPET). Secondary outcomes include peak power output during CPET, walking capacity, cognitive performance, HRQoL, fatigue, anxiety and depressive symptoms, and blood-derived biomarkers (e.g., serum neurofilament light chain, glial fibrillary acidic protein, kynurenine pathway metabolites) related to MS pathophysiology. All outcomes are assessed at baseline and discharge after three weeks. Venous blood sampling is additionally performed immediately and two hours after the first HIIT or MCT session.

**Discussion:**

CYPRO will expand current knowledge on symptom management and rehabilitation in MS to the subpopulation of pwPPMS, and will contribute to the exploration of potential disease-modifying effects of endurance training in MS. The superiority design of CYPRO will allow deriving explicit recommendations on endurance training design in pwPPMS that can be readily translated into clinical practice.

**Trial registration:**

CYPRO has been prospectively registered at ClinicalTrials.gov on 8 February 2022 (NCT05229861).

**Supplementary Information:**

The online version contains supplementary material available at 10.1186/s12883-023-03187-6.

## Background

Multiple sclerosis (MS) is a chronic inflammatory, demyelinating, and neurodegenerative disease of the central nervous system (CNS) that globally affects approximately 2.8 million people [1, 2]. In most cases, MS initially manifests as relapsing-remitting phenotype (RRMS) and frequently transitions into secondary progressive MS (SPMS) after several years. Primary progressive MS (PPMS) is the least prevalent MS phenotype, affecting 10–15% of persons with MS (pwMS) [3]. PPMS is characterized by gradual disability worsening from disease onset [4]. Disability worsening is considered to primarily evolve from neurodegenerative aspects of the MS pathophysiology, such as neuroaxonal damage, astrocytic gliosis, and mitochondrial failure due to increased oxidative stress, all of which culminate in pronounced brain and spinal cord atrophy [3, 5]. In contrast to neuroinflammation predominating in RRMS, neurodegeneration is largely unresponsive to current disease-modifying treatment [3]. In PPMS, pharmacological treatment is limited to the humanized anti-CD20 antibody ocrelizumab. However, the benefits of ocrelizumab on disease progression and brain atrophy are diminished in older persons with PPMS (pwPPMS), or those presenting with low residual inflammation [6].

Alongside ongoing efforts to improve disease-modifying treatment in pwPPMS, optimization of complementary non-pharmacological treatment options is recognized as essential to maintain and improve the health-related quality of life (HRQoL) in pwPPMS [7]. Accordingly, the International Progressive MS Alliance called upon action, proposing symptom management and rehabilitation as one key priority area for research in progressive MS [8]. In line with the proposed goals for MS therapies, endurance training qualifies as an effective means to improve HRQoL, numerous MS symptoms, such as walking impairment or fatigue, and MS-related performance impediments, such as reduced cardiorespiratory fitness, in pwMS [8–10]. Beyond that, high-intensity interval training (HIIT) has been described to beneficially modulate systemic concentrations of blood-derived biomarkers relevant to PPMS pathophysiology. For example, an acute HIIT session reduced concentrations of serum neurofilament light chain (sNfL), serving as a surrogate marker of disease progression, neuroaxonal damage, and CNS atrophy [11, 12]. Additionally, HIIT has been shown to shift systemic concentrations of neurotoxic and pro-oxidant metabolites towards neuroprotective metabolites of the immunomodulatory and neuroactive kynurenine pathway (KP) [11, 13].

However, results have been obtained from mixed samples, dominated by RRMS phenotype. The generalizability of training designs and the desired beneficial effects of endurance training on the subpopulation of pwPPMS remains unclear, given that PPMS presents with distinct clinical features, such as progressive spastic paraparesis as a hallmark symptom [14, 15]. Owing to the high prevalence of progressive spastic paraparesis, particularly pwPPMS may be prone to physical deconditioning, which is indicated by low cardiorespiratory fitness. As cardiorespiratory fitness is associated with HRQoL, walking impairment, cognitive performance, fatigue, and potential CNS tissue sparing in pwMS, improvement of cardiorespiratory fitness represents a key research outcome in MS trials and constitutes a central target in MS rehabilitation [10, 16].

With the randomized controlled trial CYPRO (CYcling in Primary PROgressive Multiple Sclerosis), we present a novel approach to validate the established mixed-sample effects of HIIT and moderate-intensity continuous training (MCT) on cardiorespiratory fitness and a comprehensive set of further MS-relevant outcomes in a sample that is exclusively composed of pwPPMS.

## Methods

This manuscript of a study protocol was designed in accordance with the Standard Protocol Items: Recommendations for Interventional Trials (SPIRIT) 2013 Statement, the Template for Intervention Description and Replication (TIDieR) checklist, and the World Health Organization’s Trial Registration Data Set (Version 1.3.1) [17–19]. The completed SPIRIT and TIDieR checklists are provided as Supplementary material 1 and Supplementary material 2. 

### Aim, study design, and setting

CYPRO is a parallel-group, single-center, and single-blind randomized controlled superiority trial that is performed at the Valens rehabilitation clinic, Switzerland.

The aims of CYPRO are described as the following:

1. Primary aim: To investigate the effects of HIIT compared to MCT on cardiorespiratory fitness, as indicated by peak oxygen consumption (V̇O_2peak_).

2. Secondary aims: To investigate the effects of HIIT compared to MCT on peak power output (PPO), walking capacity, cognitive performance, HRQoL, fatigue, anxiety and depressive symptoms, and blood-derived biomarkers related to MS pathophysiology. Additionally, CYPRO aims to investigate the acute effects of a single HIIT session compared to a single MCT session on changes in blood-derived biomarkers.

**Fig. 1:**
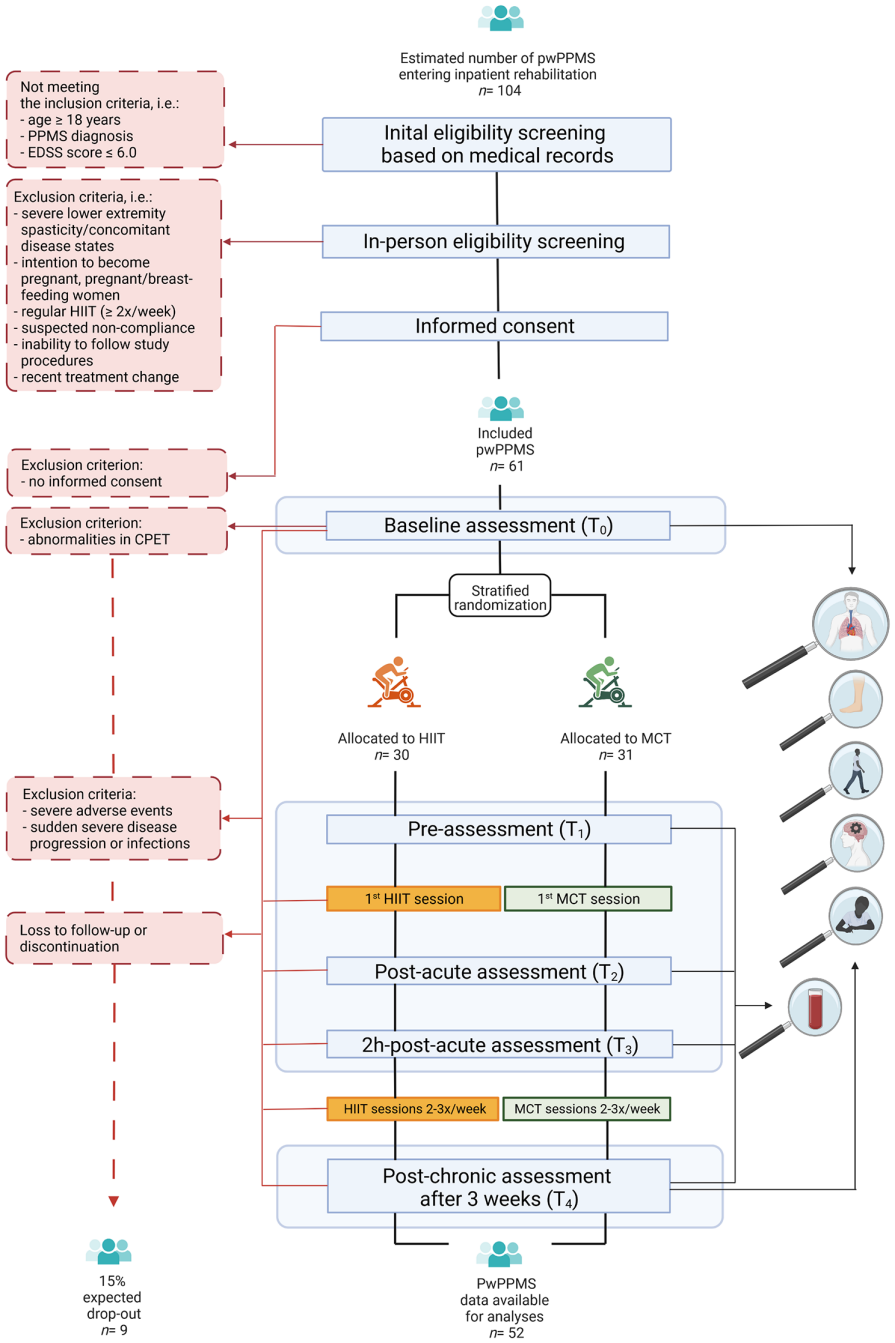
Study design and flow of participants Abbreviations: PPMS= primary progressive multiple sclerosis; pwPPMS= persons with PPMS; HIIT: high-intensity interval training; MCT: moderate-intensity continuous training; EDSS= Expanded Disability Status Scale; CPET= cardiopulmonary exercise testing. Created with BioRender.com

HIIT represents the experimental condition that is hypothesized to be superior to the standard treatment MCT as an active comparator. The study design and flow of participants are illustrated in Fig. 1.

### Sample size calculation

Sample size calculation for the estimated effect of HIIT or MCT on the primary outcome V̇O_2peak_ was performed using G*Power software (Version 3.1.9.7, Heinrich Heine Universität, Düsseldorf, Germany) [20]. Estimating a drop-out of 15%, a total of 61 participants (HIIT: *n*= 30, MCT: *n*= 31) was found to be sufficient to identify a small to moderate effect (*ES* ≥ 0.15) in ANOVA analysis on 2 group (HIIT vs. MCT) * 2 time (T_0_ vs. T_4_) interaction. Power was set at 80% and alpha at 0.05. Correlation among repeated measures was set at 0.6.

### Participants

Inclusion criteria for CYPRO comprise adult age (≥ 18 years), definite PPMS diagnosis, Expanded Disability Status Scale (EDSS) score  ≤ 6.0, and signed consent [21–23]. Exclusion criteria concern severe lower extremity spasticity or concomitant disease states (i.e., orthopaedic, cardiovascular, metabolic, psychiatric, other neurological, serious medical conditions) that would impair the ability to participate. Pregnant or breastfeeding women or those intending to become pregnant are excluded. Further, pwPPMS are prohibited to participate, if they regularly perform HIIT (i.e., ≥ 2 times per week), if non-compliance is suspected, if they are not able to follow study procedures (e.g., due to insufficient German literacy), or in case of a recent treatment change (e.g., change in disease-modifying treatment (≤ 6 weeks), stem cell treatment (≤ 6 months)). PwPPMS are excluded from further participation in case of severe adverse events (e.g., cardiovascular decompensation) during cardiopulmonary exercise testing (CPET) at baseline, sudden severe disease progression, or falling sick (e.g., due to bacterial or viral infections) during the participation in CYPRO.

### Recruitment and randomization procedure

All pwMS entering the Valens rehabilitation clinic for an inpatient stay are screened for study eligibility. Eligibility screening is performed as a two-step process. In the first step, PPMS diagnosis and EDSS score are checked in advance of clinic admission. In the second step, all other eligibility criteria are checked by in-person consultation on the day of clinic admission. Sixty-one pwPPMS will be recruited. As the sex ratio in pwPPMS is 1:1, we pursue recruiting an equal number of female and male pwPPMS [14]. The number of screened pwMS in total and per screening step, as well as reasons for ineligibility are documented.

Stratified randomization to either HIIT or MCT (1:1) is performed by a group of blinded investigators not involved in CYPRO. Allocation sequence generation is performed using random-sized (block size 4–6) permuted block randomization with Randomization-In-Treatment-Arms (RITA) software (Version 1.51, Evident, Lübeck, Germany). To ensure allocation concealment, the blinded investigators are called for assignment each time a participant presents for inclusion [24]. CYPRO study personnel that performs enrolment, obtains consent, and assigns participants has no access to details on the allocation sequence and blocking. Strata include relative V̇O_2peak_ (mL · min^-1^ · kg-^-1^) at baseline, sex, age, and EDSS score.

### Exercise protocols

Within their three-week inpatient stay at the Valens rehabilitation clinic, participants perform two to three weekly HIIT or MCT sessions on bicycle ergometers (Cybex 750 C, Cybex International Inc., Massachusetts, USA) in addition to standard rehabilitative care. Standard rehabilitative care comprises endurance and strength training (30-45 minutes, three to five times per week), physiotherapy to improve balance and/or walking ability (30 minutes, daily) as well as occupational therapy focused on fatigue management and activities of daily living (30 minutes, two to three times per week). Exercise volume of HIIT and MCT protocols have been matched in Units of Exercise. Units of Exercise are calculated as [intensity (percentage of peak heart rate (%HR_peak_)) x session duration (minutes) x frequency (number of sessions per week) x number of weeks] [25]. %HR_peak_ is derived from HR_peak_ achieved during CPET at baseline. During sessions, HR is continuously recorded by HR sensors (H10 HR sensor, POLAR, Kempele, Finland) attached to chest belts, and connected to wristwatches (M430 sports watch, POLAR, Kempele, Finland). Both, HIIT and MCT are conducted under the supervision of trained exercise scientists and physiotherapists, either individually or in small groups of up to three participants. Therapists are instructed to monitor the HR, and to adjust pedaling resistance in case of deviations from the target intensity. If participants are unable to follow the prescribed protocols (e.g., due to pronounced ankle flexor spasticity), dose modifications (i.e., decreasing revolutions per minute (rpm) or interval duration, increasing break duration) are permissible. Any dose modification is documented in a case report form. Blinding of therapists and participants towards group allocation is not feasible due to the study design.

#### High-intensity interval training (HIIT)

HIIT sessions commence with a two-minute low-intensity warm-up (60%HR_peak_) at 60-70 rpm. Subsequently, six 90-second high-intensity intervals (95%HR_peak_) are performed at high pedaling rates of 80-100 rpm. Intervals are interspersed by 90-second active breaks with unloaded pedaling at 60-70 rpm, aimed to return to 60%HR_peak_. Sessions close with a two-minute low-intensity (60%HR_peak_) cool-down of unloaded pedaling at 60-70 rpm. In total, one HIIT session lasts 21 minutes.

#### Moderate-intensity continuous training (MCT)

MCT represents the standard treatment at Valens rehabilitation clinic and is used as an active comparator. Participants perform continuous bicycle ergometry at moderate intensity (60%HR_peak_) and pedaling rates of 60-70 rpm for the duration of 26 minutes.

#### Safety considerations

Exercise is safe in pwMS and not associated with a higher risk of adverse events compared to exercise in healthy individuals [26]. Similarly, HIIT on bicycle ergometers is well-tolerated and holds a low risk of adverse events in pwMS [27]. As in healthy individuals, transient knee and/or leg pain, or muscle soreness may occur in response to HIIT and MCT sessions. Between HIIT and MCT sessions, participants receive at least 48 hours of rest to ensure adequate recovery. Supervising therapists are instructed to prevent, monitor, and document the occurrence of adverse events, if any. To prevent injuries or falls, participants may be assisted in getting on and off the bicycle ergometer, if necessary. HR monitoring, rating of perceived exertion (RPE, Borg Category Ratio-10-point (Borg CR-10) scale), and observation of vegetative signs serve to minimize any risk of overexertion [28]. Study personnel may need to withdraw participants in case of repeatedly reported adverse events (e.g., leg pain), the occurrence of severe adverse events (e.g., cardiovascular decompensation), or other medical reasons (e.g., infections). In those cases, participants are forwarded to a physician for medical clearance. With the signature of the consent form prior to participation, participants confirm that they are informed about potential exercise-related risks and the right to refrain from participation in CYPRO at any time.

### Outcomes

V̇O_2peak_, PPO, walking capacity, cognitive performance, HRQoL, fatigue, and anxiety and depressive symptoms are assessed by allocation-blinded outcome assessors at study entry (baseline, T_0_) and discharge after three weeks (T_4_), at least 48 hours after the last HIIT or MCT session. Venous blood samples are taken before (T_1_), immediately after (T_2_), and two hours after (T_3_) the first HIIT or MCT session, and at T_4_.

#### Baseline variables

Demographic data (sex, age) and MS-related data (EDSS score, time since diagnosis (months), pharmacological treatment, if any) are obtained from medical records. Bodyweight is determined by digital scales (Soehnle Style Sense Comfort 100, Soehnle, Nassau, Germany) at fasted state without footwear. Body weight (kg) and self-reported body height (cm) are used to calculate the body mass index (BMI). Cognitive status at baseline is evaluated using the Montreal Cognitive Assessment (MoCA). The MoCA is 30-point test used to assess global cognitive status, including items addressing short-term memory, visuospatial abilities, executive functions, attention, concentration, working memory, language, and orientation. A MoCA score < 26 indicates cognitive impairment [29]. Sensitivity and specificity to discriminate between cognitively impaired and non-impaired pwMS have been proven [30]. MoCA performance does not influence the eligibility to participate in CYPRO.

#### Cardiorespiratory fitness

V̇O_2peak_ is assessed by CPET on a bicycle ergometer (ergometrics er800s, ergoline GmbH, Bitz, Germany). CPET is performed in a fasted state between 8:00 and 9:00 AM and follows a ramp-type protocol. The ramp-type protocol consists of (a) a resting state measurement without pedaling while participants are sitting on the bicycle ergometer (3 minutes); (b) subsequent pedaling at 20 watts (3 minutes); (c) the testing phase with a progressive increment of 5 to 10 watts per minute until subjective exhaustion (8-12 minutes); (d) followed by a cool-down of unloaded pedaling (3 minutes). HR is continuously monitored. Blood pressure and RPE (Borg CR-10 scale) are assessed every two minutes and within the last ten seconds of the test.

V̇O_2_ is monitored by direct and continuous measurements (breath by breath) by ergospirometry (Vyaire Medical, Vyntus CPX, Illinois, USA). V̇O_2peak_ is defined as the highest 15-second averaged V̇O value when the following criteria are attained: respiratory equivalent ratio > 1.10; HR_peak_ within 10 min^−1^ of the age-predicted maximum and Borg-CR-10 rating > 8.5 [31]. The absolute V̇O_2peak_ value (mL · min^−1^) is divided by bodyweight (kg) to obtain the relative V̇O_2peak_ value (mL · min^− 1^ · kg^− 1^) as the primary outcome.

#### Peak power output (PPO)

PPO is assessed as the peak wattage achieved during CPET and represents the maximum mechanical power produced by lower extremity musculature. PPO is considered a measure of the physical functional reserve. In pwMS, lower PPO is correlated with higher energetic costs of walking [32].

#### Walking capacity

Walking capacity is tested using the six-minute walk test (6-MWT). Participants are asked to walk back and forth on a 30-meter hallway for the duration of six minutes, performing 180° turns around cones at each end [33]. According to the modified 6-MWT script, participants are instructed to walk at maximum speed. Breaks or any kind of encouragement are not permitted. Participants are allowed to use an assistive walking device, if necessary to ensure safe ambulation. 6-MWT performance is defined as the total distance in meters covered within six minutes. The modified 6-MWT has excellent inter-rater and intra-rater reliability, and correlates with fatigue, self-reported physical functionality, and perceived ambulation impairment in pwMS [34].

#### Cognitive performance

Cognitive performance is tested using the validated German modification of the Brief International Cognitive Assessment for Multiple Sclerosis (BICAMS-M). Similar to the original BICAMS, this modified version comprises three subtests evaluating information processing speed, verbal memory, and visuospatial memory [35]. The Symbol Digit Modalities Test (SDMT) evaluates information processing speed. The participant is displayed a code of nine abstract symbols, paired with nine digits. Underneath the code, incomplete rows are presented that only contain the abstract symbols in a pseudo-random order. The participant is asked to verbally match as many digits as possible to the corresponding abstract symbol within 90 seconds. SDMT performance is indicated by the number of correct matches, written down by the outcome assessor. Instead of the California Verbal Learning Test, the German language Verbal Learning and Memory Test (VLMT) was adopted, as norm data are based on a larger validation cohort. The VLMT is used to assess verbal memory. Participants are read aloud a 15-word list five times. After each repetition, participants are asked to immediately recall as many words as possible. VLMT performance is indicated by the summed number of correct words across the five trials. The Brief Visuospatial Memory Test-Revised (BVMT-R) is performed to assess visuospatial memory. The participant is asked to memorize six abstract geometric figures, presented on a 2 × 3 array for ten seconds. After these ten seconds, the array is removed. The participant is asked to copy the six figures on a blank form. The procedure is repeated three times. For each trial, the outcome assessor rates the shape, size, and location of the figures on a 0-2 scale. Higher ratings indicate better performance. Overall BVMT-R performance is indicated by the summed number of ratings across the three trials [36, 37]. Parallel versions of the VLMT and the BVMT-R are used at T_4_. For the SDMT, no parallel version is used as learning effects are considered to be minor [37, 38]. The validated BICAMS-M allows to detect cognitive impairment in MS and is a reliable instrument to monitor cognitive performance over time [37].

#### Health-related quality of life

HRQoL is assessed using the German version of the Multiple Sclerosis Impact Scale (MSIS-29). The MSIS-29 comprises two subscales, addressing the physical impact (20 items), and psychological impact (9 items) of MS. Items address upper and lower limb functionality, ambulation, incontinence, sleep, emotional well-being as well as disease-related limitations in daily living, and societal participation. Each item is ranked on a 5-point Likert scale. Higher scores indicate a greater impact of MS and lower HRQoL. To calculate subscale scores, individual scores are summed, averaged, and transformed to a 0-100 scale [39, 40]. The German version additionally allows to calculate a total MSIS-29 score that is the arithmetic mean of both subscale scores [40, 41]. The MSIS-29 is a responsive measure in MS rehabilitation, and has been used in pwPPMS [42]. The German version of the MSIS-29 has been proven to be valid and reliable [40, 41].

#### Fatigue

MS-related fatigue is assessed with the German version of the Fatigue Scale for Motor and Cognitive Functions (FSMC). The FSMC is a multidimensional 20-item composite scale that comprises a 10-item motor and a 10-item cognitive subscale. Items are rated on a 5-point Likert scale. Higher values indicate greater total, motor, or cognitive fatigue, respectively. Cut-off values allow to identify substantial fatigue (i.e., FSMC composite score ≥ 43), and to classify fatigue severity as mild, moderate, or severe. The FSMC composite scale as well as both subscales have been proven to be reliable, and are sensitive and specific for detecting MS-related fatigue [43].

#### Anxiety and depressive symptoms

Anxiety and depressive symptoms are assessed with the validated German version of the Hospital Anxiety and Depression Scale (HADS). The HADS is used in non-psychiatric populations with medical ailments, and includes 14 items that allow evaluation of anxiety (7 items) and depressive symptoms (7 items). Items are scored on a 4-point Likert scale. According to the original HADS manuscript, separate sum scoring is performed for the items on anxiety and depressive symptoms. Higher values indicate greater severity of anxiety or depressive symptoms. Subscale score ranges are used to distinguish between non-cases (0-7 points), doubtful cases (possible anxiety/depression, 8-10 points), and cases (probable anxiety/depression, 11-21 points) [44, 45]. A HADS total score of ≥ 13 points is considered to indicate overall psychological distress [46, 47]. For the German population, normative values are available [46]. In pwMS, the HADS is a sensitive and specific self-report measure that supports the detection of major depression, and/or generalized anxiety disorder [48].

#### Blood-derived biomarkers

Blood sampling is performed in a fasted state. Samples are obtained from the antecubital vein in supine position. To evaluate the chronic effects of HIIT and MCT on blood-derived biomarkers, resting blood samples are taken between 8:00 and 9:00 AM after ten minutes of supine rest (T_1_, T_4_). To evaluate acute effects on blood-derived biomarkers, blood samples are taken immediately after (T_2_) and two hours after (T_3_) the first HIIT or MCT session. Per sampling time point, two whole blood collection tubes (BD Vacutainer^®^, BD CPT™, 4ml), prefilled with lymphocyte separation medium, and one serum tube (tube vacutainer SSTII serum yel, 6ml) are taken.

Blood samples are analysed regarding the acute and chronic effects of HIIT and MCT on KP modulation, and surrogate markers of neurodegeneration (i.e., sNfL, glial fibrillary acidic protein (GFAP)).

Whole blood samples are centrifuged at 3500 g for 20 minutes, separating peripheral blood mononuclear cells (PBMCs) and plasma. PBMCs are resuspended in plasma, purged into centrifugation tubes, diluted with an equal amount of phosphate-buffered saline, and centrifuged at 2400 g for 10 minutes. The supernatant is discarded. PBMCs are resuspended in cell culture freezing medium, and aliquoted. Aliquots are frozen at -80 °C until analysis. mRNA of PBMCs is isolated using a commercial column-based isolation kit. cDNA synthesis is performed. Based on mRNA and cDNA, gene expression of KP-relevant genes is determined. As such, expression levels of indoleamine 2,3-dioxygenase-1, kynurenine aminotransferase 1–4, kynurenine-3-monooxygenase, aryl hydrocarbon receptor, CYP1A1, interleukin-4-induced-1, and SLC7A5 are determined using real-time quantitative polymerase chain reaction (qPCR), run on a qTower³ G touch (Analytik Jena GmbH, Jena, Germany). Blood serum is centrifuged at 2500 g for 20 minutes, is aliquoted, and frozen at -80 °C until analysis. Targeted metabolomics (liquid chromatography tandem mass spectrometry (LC-MS/MS)) is performed at BEVITAL AS, Bergen, Norway, to determine serum concentrations of tryptophan, KP downstream metabolites (e.g., kynurenine, kynurenic acid, quinolinic acid), and B vitamers. Systemic concentrations of sNfL and GFAP are assessed using a single molecule array (SiMoA HD-1 device, Quanterix, USA) at the University Medical Center of the Johannes Gutenberg University, Mainz, Germany, according to the manufacturer’s instructions.

**Fig. 2:**
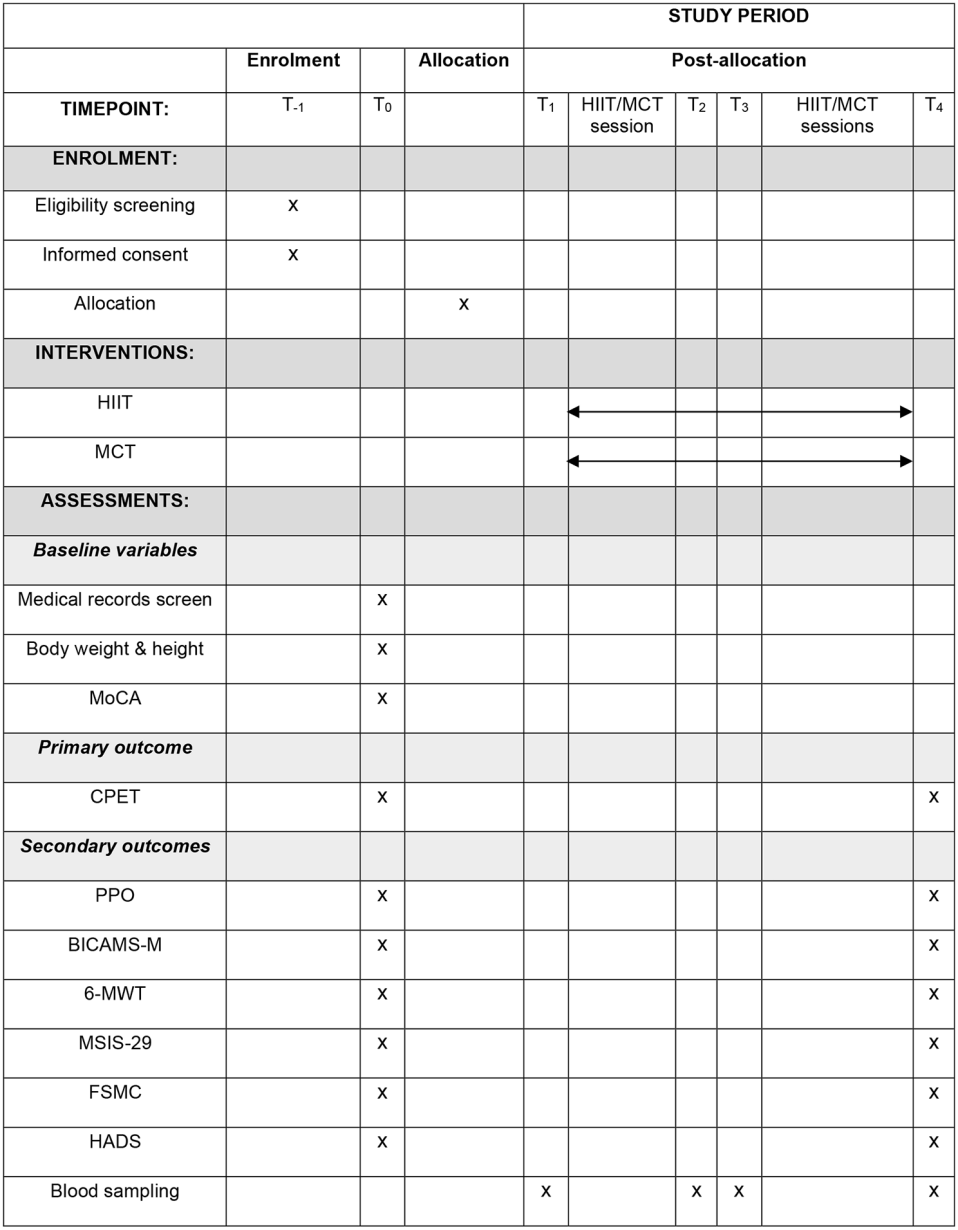
SPIRIT 2013 diagram Abbreviations: HIIT = high-intensity interval training; MCT = moderate-intensity continuous training; MoCA = Montreal Cognitive Assessment; CPET = cardiopulmonary exercise testing; PPO = peak power output in watts during CPET; BICAMS-M = German modification of the Brief International Cognitive Assessment for Multiple Sclerosis; 6-MWT = six-minute walk test; MSIS-29 = Multiple Sclerosis Impact Scale; FSMC = Fatigue Scale for Motor and Cognitive Functions; HADS = Hospital Anxiety and Depression Scale. T_− 1_= day of admission; T_0_ = day of study entry (baseline assessment prior group allocation); T_1_ = immediately before the first exercise session; T_2_ = immediately after the first exercise session; T_3_ = two hours after the first exercise session; T_4_ = at discharge after three weeks, at least 48 h after the last exercise session

Sequences and time frames of assessment procedures are depicted in the SPIRIT 2013 diagram (Fig. 2).

#### Compliance

Drop-out and session attendance as well as reasons for study withdrawal and incomplete session attendance are captured in total, and separately for HIIT and MCT. Participants who drop out continue standard rehabilitative care at the Valens rehabilitation clinic, as far as medical conditions allow. Participants are not replaced. Collected data are stored upon the termination of data analysis. None of the assessments planned at later stages are conducted. The attendance rate is calculated as the number of completed sessions by the number of prescribed sessions. Protocol adherence to the prescribed duration and intensity is derived from HR recordings of HIIT and MCT sessions upon completion of data collection. Reasons for session abortion or protocol deviations, including but not limited to dose modifications and adverse events, are questioned, and documented in a case report form. Severe adverse events (e.g., cardiovascular decompensation) are directly reported to the local Ethics committee. Overall compliance is assessed by comparing prescribed Units of Exercise to performed Units of Exercise per group, combining measures of adherence (intensity (%HR_peak_), session duration (minutes)), and attendance (total number of sessions, i.e., number of sessions per week x number of weeks) [25]. Compliance will be given as percentage of prescribed Units of Exercise.

### Data management and confidentiality

Data generation, transmission, storage, and analysis follow Swiss legal requirements for data protection. Personal data are considered confidential and disclosure to third parties is prohibited. The anonymity of participants is guaranteed by utilizing unique subject identification code numbers that are consecutively generated by a computerized list. Personal data and the allocation list are locked separately from anonymized baseline variables and outcome data. Unblinding of outcome assessors towards allocation is permissible upon completion of T_4_. Data of all outcomes as well as case report forms will be archived (1) in folders that are stored in locked study closets, and (2) electronically on personalized password-secured desktop computers. Backups are automatically performed every hour. Access is provided to authorized study personnel, the Clinical Trial Board of the Kliniken Valens hospital group, and the local Ethics committee at all times for purposes of trial-related monitoring, audits, review, and regulatory inspections.

### Statistical analysis

Statistical analyses are performed according to the intention-to-treat principle. Descriptive statistics will be reported as arithmetic mean (*M*) and standard deviation (*SD*) for continuous data, and as absolute number and percentage (*%*) for categorical data for the total sample, and separated by group. Outcome data will be checked for normality (Shapiro-Wilk test) in advance. If necessary, analyses will be adjusted accordingly. If missing values are < 5%, ANCOVA will be performed to detect time*group interaction effects and main effects of time. In that case, effect sizes will be calculated as partial eta squared (*pη*^*2*^). Otherwise, a baseline-adjusted Mixed Model for Repeated Measures (MMRM) approach, with group, time and the group × time interaction as fixed effects (type III sums of squares, CS covariance structure over time) is used to assess between-group differences over time. Bonferroni-corrected pairwise comparisons of estimated marginal means of the group × time interaction are computed. Effect sizes will be reported as Cohen’s *d* with 95% confidence interval (*CI*). For all analyses, the level of significance is set at *p* = .05. Within-group differences and between-group differences are depicted as point estimates and measures of variability for all outcomes. Bivariate correlation analyses will be conducted to determine potential associations between changes in V̇O_peak_, PPO, walking capacity, cognitive performance, HRQoL, fatigue, anxiety and depressive symptoms, and blood-based biomarkers using Pearson’s *r*. All statistical analyses will be conducted with IBM SPSS Statistics for Windows (Version 29.0., IBM Corp., Armonk, New York, USA). Besides the final analysis, one interim analysis will be performed upon collection of 50% of participant data (HIIT: *n* = 15, MCT: *n* = 16).

## Discussion

CYPRO primarily aims to evaluate the effects of two different endurance training modalities, that are HIIT and MCT, on cardiorespiratory fitness in pwPPMS, representing the rarest and least investigated MS phenotype [3]. Using a comprehensive set of outcome measures, that combine performance indices (i.e., V̇O_2__peak_, PPO, walking capacity, cognitive performance), patient-reported outcome measures on HRQoL, fatigue, anxiety and depressive symptoms, and blood-derived biomarkers, CYPRO will expand current knowledge on the effects of endurance training in MS to the subpopulation of pwPPMS. Thus, CYPRO is a direct response to the International Progressive MS Alliance’s call for action to “expedite the development of disease-modifying and symptom-relief treatments for progressive MS”, by focusing on the key priority research area of symptom management and rehabilitation [8].

Among candidate non-pharmacological treatment options, endurance training is a powerful means to improve HRQoL, numerous MS symptoms, and MS-related performance impediments [9, 10]. Meanwhile, not only MCT but also HIIT found incorporation in current treatment recommendations [49, 50]. HIIT is safe and feasible, and may be a more enjoyable endurance training option than MCT for pwMS [27]. Moreover, HIIT revealed to be superior to MCT in improving V̇O_peak_ and cognitive performance in pwMS and has been shown to beneficially modulate concentrations of blood-derived biomarkers relevant to MS pathophysiology, such as sNfL [11, 51, 52]. Those results have been obtained from previous studies that have been performed in the same setting, and involved HIIT and MCT protocols similar to those designed for CYPRO. Under the premise that pwPPMS respond similarly to HIIT and MCT as mixed samples, findings may be expected to be reproduced.

As a prospective study that is well-powered for pwPPMS, CYPRO is a novel approach accounting for the distinct features of pwPPMS, that besides PPMS-relevant performance indices and patient-reported outcomes measures, includes blood-derived biomarkers, such as sNfL or GFAP, that are closely related to neurodegenerative aspects of PPMS pathophysiology. Herewith, CYPRO will not only expand knowledge on symptom management and rehabilitation, but will also contribute to the exploration of potential disease-modifying effects of endurance training in MS. The superiority design of CYPRO will allow deriving explicit recommendations on endurance training design in pwPPMS that can be readily translated into clinical practice.

## Study status

The first participant was enrolled on 13 March 2022. Participants are currently being recruited. Until March 2023, 260 pwMS entering the Valens rehabilitation clinic have been screened for study eligibility. Fifty-five pwMS held PPMS diagnosis. Among those, 23 pwPPMS have been recruited for CYPRO.

## Publication and dissemination

Findings of CYPRO are condensed in manuscripts according to the Consolidated Standards of Reporting Trials (CONSORT) 2010 Statement and will be published in pertinent peer-reviewed journals [53]. No professional writers will be involved. Further, findings will be presented at relevant congresses and will be disseminated to the participants, relevant expert groups, and the public. The Swiss Multiple Sclerosis Society receives a final report.

## Electronic supplementary material

Below is the link to the electronic supplementary material.

Supplementary Material 1

Supplementary Material 2

## Data Availability

The full protocol submitted to the Ethics committee, the charter of the Clinical Trial Board, model consent forms, data collection forms, case report forms, details of data management procedures, and datasets used and/or analyzed during the current study are available from the corresponding author upon reasonable request. Current license rights for testing manuals, questionnaires, and scales apply. Sharing of deidentified individual clinical trial participant-level data (IPD) is not intended.

## Abbreviations

BICAMS-M: German modification of the Brief International Cognitive Assessment for Multiple Sclerosis
BMI: Body mass index
Borg CR-10 scale: Borg Category Ratio-10-point scale
BVMT-R: Brief Visuospatial Memory Test-Revised
CNS: Central nervous system
CONSORT: Consolidated Standards of Reporting Trials
CPET: Cardiopulmonary exercise testing
CYPRO: CYcling in primary PROgressive multiple sclerosis
EDSS: Expanded Disability Status Scale
EKOS: Ethikkommission Ostschweiz
FSMC: Fatigue Scale for Motor and Cognitive Functions
GFAP: Glial fibrillary acidic protein
HADS: Hospital Anxiety and Depression Scale
HIIT: High-intensity interval training
HRQoL: Health-related quality of life
HR_peak_: Peak heart rate during cardiopulmonary exercise testing
KP: Kynurenine pathway
LC-MS/MS: Liquid chromatography tandem mass spectrometry
MMRM: Mixed Model for Repeated Measures
MCT: Moderate-intensity continuous training
MoCA: Montreal Cognitive Assessment
MS: Multiple sclerosis
MSIS-29: Multiple Sclerosis Impact Scale
PBMC: Peripheral blood mononuclear cells
PPMS: Primary progressive multiple sclerosis
PPO: Peak power output (peak wattage during cardiopulmonary exercise testing)
pwMS: Persons with multiple sclerosis
pwPPMS: Persons with primary progressive multiple sclerosis
qPCR: Quantitative polymerase chain reaction
RITA: Randomization-In-Treatment-Arms
RRMS: Relapsing-remitting multiple sclerosis
RPE: Rating of perceived exertion
rpm: Revolutions per minute
SDMT: Symbol Digit Modalities Test
SPIRIT: Standard Protocol Items: Recommendations for Interventional Trials
SPMS: Secondary progressive multiple sclerosis
SiMoA: Single molecule array
sNfL: Serum neurofilament light chain
TIDieR: Template for Intervention Description and Replication
VLMT: Verbal Learning and Memory Test
V̇O_2__peak_: Peak oxygen consumption during cardiopulmonary exercise testing
6-MWT: Six-minute walk test
