# Supplementary material for: Cycling in primary progressive multiple sclerosis (CYPRO): study protocol for a randomized controlled superiority trial evaluating the effects of high-intensity interval training in persons with primary progressive multiple sclerosis
Source: BMC Neurol. 2023 Apr 22;23:162. doi: 10.1186/s12883-023-03187-6 (PMC10122389; doi:10.1186/s12883-023-03187-6)
Supplement: Supplementary file 2 — Supplementary Material 2 [file 12883_2023_3187_MOESM2_ESM.pdf]

Additional file 2 TIDieR checklist

Intervention: High-intensity training (HIIT) on bicycle ergometers

**Why:**	HIIT revealed to be superior to moderate-intensity continuous training (MCT) in improving cardiorespiratory fitness, measured as peak oxygen consumption ($\dot{V}O_{2peak}$) and cognitive performance and has been shown to beneficially modulate concentrations of blood-derived biomarkers relevant to the pathophysiology of primary progressive multiple sclerosis (PPMS), such as serum neurofilament light chain.
**What (material):**	All participants provided written informed consent. Participants perform HIIT sessions on bicycle ergometers (Cybex 750C, Cybex International Inc., Massachusetts, USA). During sessions, heart rate (HR) is continuously recorded by HR sensors (H10 HR sensor, POLAR, Kempele, Finland) attached to chest belts, and connected to wristwatches (M430 sports watch, POLAR, Kempele, Finland). Any dose modification, reasons for session abortion or protocol deviations, including but not limited to necessary dose modifications and adverse events, are questioned and documented in the case report form.
**What (procedures):**	Within their three-week inpatient stay at Valens rehabilitation clinic, participants perform HIIT sessions in addition to standard rehabilitative care. Standard rehabilitative care includes endurance and strength training sessions (30–45 min, three to five times per week), physiotherapy sessions to improve balance and/or walking ability (30 min daily) as well as occupational therapy (30 min, two to three times per week) focused on fatigue management and activities of daily living. Exercise volume of HIIT and MCT protocols have been matched in “Units of Exercise”, calculated as [intensity (%HR_{peak}) x duration (minutes per session) x frequency (sessions per week) x (number of weeks). Exercise intensity is derived from HR_{peak} achieved during cardiopulmonary exercise testing (CPET) at baseline and calculated as percentage HR_{peak} (%HR_{peak}). During sessions, HR is continuously recorded.
**Who provides:**	HIIT sessions are supervised by trained exercise scientists and physiotherapists. Therapists are instructed to prevent injuries or falls and may assist participants in getting on and off the bicycle ergometer, if necessary. Therapists are instructed to monitor the HR, and to adjust pedaling resistance in case of deviations from the target intensity. Therapists are educated to inquire the rate of perceived exertion (RPE, Borg Category Ratio-10-point (Borg CR-10) scale), and to observe vegetative signs to minimize any risk of overexertion.
**How (mode of delivery; individual or group):**	HIIT sessions are performed during participants' inpatient stay and are supervised by trained exercise scientists and physiotherapists individually, or in small groups of up to three participants.
**Where:**	HIIT sessions are performed at Valens rehabilitation clinic, Switzerland.
**When and how much:**	Within their three-week inpatient stay at Valens rehabilitation clinic, participants perform two to three weekly HIIT sessions. HIIT sessions commence with a two-minute low-intensity warm-up (60%HR_{peak}) at 60-70 revolutions per minute (rpm). Subsequently, six 90-second high-intensity intervals (95%HR_{peak}) are performed at high pedaling rates of 80-100 rpm. Intervals are interspersed by 90-second active breaks with unloaded pedaling (20 watts) at 60-70 rpm, aimed to return to 60%HR_{peak}. Sessions close with a two-minute low-intensity (60%HR_{peak}) cool-down of unloaded pedaling at 60-70 rpm. In total, one HIIT session lasts 21 minutes.
**Tailoring:**	Yes. Tailoring of exercise intensity is performed by deriving from HR_{peak} from baseline CPET and calculating percentage HR_{peak} (%HR_{peak}) for HIIT sessions as described in paragraph 7. If participants are unable to follow the prescribed protocols (e.g., due to pronounced ancle plantar flexor spasticity), dose modifications (i.e., decreasing revolutions per minute (rpm) or interval duration, increasing break duration) are permissible. Adaption of the HIIT protocol is not intended due to the limited intervention duration of three weeks.
**How well (planned):**	Drop-out and session attendance as well as reasons for study withdrawal and incomplete attendance are captured in total, and separately for HIIT and MCT. The attendance rate is calculated as the number of completed sessions by the number of prescribed sessions. Protocol adherence to the intended duration and intensity is derived from HR recordings of HIIT sessions upon completed data collection. Reasons for session abortion or protocol deviations, including but not limited to necessary dose modifications and adverse events, are questioned, and documented in the case report form. Overall compliance is assessed by comparing prescribed Units of Exercise to performed Units of Exercise per group, combining measures of adherence (intensity (%HR_{peak}), duration per session (minutes)), and attendance (total number of sessions, i.e., number of sessions per week x number of weeks). Compliance will be given as % of prescribed Units of Exercise.

Comparison/Control: Moderate-intensity continuous training (MCT) on bicycle ergometers

**Why:**	MCT is the standard treatment at Valens rehabilitation clinic, Switzerland.
**What (material):**	All participants provided written informed consent. Participants perform MCT sessions on bicycle ergometers (Cybex 750C, Cybex International Inc., Massachusetts, USA). During sessions, HR is continuously recorded by HR sensors (H10 HR sensor, POLAR, Kempele, Finland) attached to chest belts, and connected to wristwatches (M430 sports watch, POLAR, Kempele, Finland). Any dose modification, reasons for session abortion or protocol deviations, including but not limited to necessary dose modifications and adverse events, are questioned and documented in the case report form.
**What (procedures):**	Within their three-week inpatient stay at Valens rehabilitation clinic, participants perform MCT sessions in addition to standard rehabilitative care. Standard rehabilitative care includes endurance and strength training sessions (30–45 min, three to five times per week), physiotherapy sessions to improve balance and/or walking ability (30 min daily) as well as occupational therapy (30 min, two to three times per week) focused on fatigue management and activities of daily living. Exercise volume of HIIT and MCT protocols have been matched in "Units of Exercise", calculated as [intensity (%HR_{peak}) x duration (minutes per session) x frequency (sessions per week) x (number of weeks). Exercise intensity is derived from HR_{peak} achieved during CPET at baseline and calculated as percentage HR_{peak} (%HR_{peak}). During sessions, HR is continuously recorded.
**Who provides:**	MCT sessions are supervised by trained exercise scientists and physiotherapists. Therapists are instructed to prevent injuries or falls and may assist participants in getting on and off the bicycle ergometer, if necessary. Therapists are instructed to monitor the HR, and to adjust pedaling resistance in case of deviations from the target intensity. Therapists are educated to inquire the rate of perceived exertion (Borg CR-10 scale), and to observe vegetative signs to minimize any risk of overexertion.
**How (mode of delivery; individual/group):**	MCT sessions are performed during participants' inpatient stay and are supervised by trained exercise scientists and physiotherapists individually, or in small groups of up to three participants.
**Where:**	MCT sessions are performed at Valens rehabilitation clinic, Switzerland.
**When and how much:**	Within their three-week inpatient stay at Valens rehabilitation clinic, participants perform two to three weekly MCT sessions. Participants perform continuous bicycle ergometry at moderate intensity (60%HR_{peak}) and pedaling rates of 60-70 rpm for the duration of 26 minutes.
**Tailoring:**	Yes. Tailoring of exercise intensity is performed by deriving from HR_{peak} from baseline CPET and calculating percentage HR_{peak} (%HR_{peak}) for MCT sessions as described in paragraph 7. If participants are unable to follow the prescribed protocols (e.g., due to pronounced ancle plantar flexor spasticity), dose modifications (i.e., decreasing revolutions per minute (rpm) or interval duration, increasing break duration) are permissible. Adaption of the MCT protocol is not intended due to the limited intervention duration of three weeks.
**How well (planned):**	Drop-out and session attendance as well as reasons for study withdrawal and incomplete attendance are captured in total, and separately for HIIT and MCT. The attendance rate is calculated as the number of completed sessions by the number of prescribed sessions. Protocol adherence to the intended duration and intensity is derived from HR recordings of MCT sessions upon completed data collection. Reasons for session abortion or protocol deviations, including but not limited to necessary dose modifications and adverse events, are questioned, and documented in the case report form. Overall compliance is assessed by comparing prescribed Units of Exercise to performed Units of Exercise per group, combining measures of adherence (intensity (%HR_{peak}), duration per session (minutes)), and attendance (total number of sessions, i.e., number of sessions per week x number of weeks). Compliance will be given as % of prescribed Units of Exercise.
